# Prevalence of enteroviruses in children with and without hand, foot, and mouth disease in China

**DOI:** 10.1186/1471-2334-13-606

**Published:** 2013-12-27

**Authors:** Xiaoai Zhang, Hongyu Wang, Shujun Ding, Xianjun Wang, Xiaodan Chen, Ying Wo, Liyuan Wang, Doudou Huang, Wei Liu, Wuchun Cao

**Affiliations:** 1 Graduate School of Anhui Medical University, 230032, Hefei, P. R. China; 2 State Key Laboratory of Pathogen and Biosecurity, Beijing Institute of Microbiology and Epidemiology, 20 Dong-Da Street, Fengtai District, Beijing 100071, P. R. China; 3 Shandong Provincial Center for Disease Control and Prevention, 250001, Jinan, P. R. China

**Keywords:** Hand, foot, and mouth disease, Enterovirus, Epidemiology

## Abstract

**Background:**

To determine the prevalence of human enteroviruses (HEVs) among healthy children, their parents, and children with hand, foot, and mouth disease (HFMD).

**Methods:**

We conducted a case–control study that included throat samples from 579 children with HFMD and from 254 healthy controls. Throat samples from 49 households (98 parents and 53 healthy children) were also analyzed. Phylogenetic analysis was carried out to study genetic relationships of EV71 strains.

**Results:**

The HEV positive rate in HFMD patients was significantly higher than that in healthy controls (76.0% vs. 23.2%, *P* < 0.001). The EV71 (43.7% vs. 15.0%, *P* < 0.001), CVA16 (18.0% vs. 2.8%, *P* < 0.001), and CVA10 (5.7% vs. 0.8%, *P* = 0.001) serotypes were significantly overrepresented in HFMD patients in comparison to healthy children. Other HEV serotypes were detected with comparable frequency in cases and controls. The HEV positive rate in severe HFMD patients was significantly higher than that in mild group (82.1% vs. 73.8%, *P* = 0.04). The EV71 (55.0% vs. 39.7%, *P* = 0.001) and CVA16 (11. 9% vs. 20.0%, *P* = 0.024) positive rate differed significantly between severe and mild HFMD patients. Other HEV serotypes were detected with comparable frequency between severe and mild HFMD patients. Among 49 households, 22 households (44.9%) had at least 1 family member positive for HEV. Children had significantly higher HEV positive rate than adult (28.3% vs. 14.3%, *P* = 0.037). The HEV positive rate was similar between mothers and fathers (12.24% vs. 16.32%, *P* = 0.56). The VP1 sequences of EV71 from HFMD patients and healthy children were nearly identical and all were clustered in the same clade, C4a.

**Conclusions:**

Our study demonstrated the co-circulation of multiple HEV serotypes in children with and without HFMD during epidemic. Our study deserves the attention on HFMD control.

## Background

Enteroviruses (EVs) are among the most common human viruses infecting humans, causing a wide spectrum of illness. On the basis of phylogenetic analysis, the genus *Enterovirus* (family *Picornaviridae*) is divided into 12 species (http://www.picornaviridae.com). Members of human enteroviruses (HEVs) include 7 species, four HEV species and 3 recently subsumed human rhinoviruses species. Although infections caused by HEVs are often asymptomatic or mild, they can cause more severe conditions, such as neurological disease, poliomyelitis, severe neonatal systemic disease, encephalitis, meningitis, or myocarditis.

Hand, foot, and mouth disease (HFMD) is a common disease caused by HEV infection among children, particularly in those less than 5-year-old. HFMD occurs worldwide epidemically, with enterovirus 71 (EV71) and coxsackievirus A16 (CVA16) taking predominant roles in causing outbreak, while other HEV serotypes were largely associated with sporadic cases. In the past decade, the size and frequency of HFMD outbreaks have greatly increased in the Asia-Pacific region, especially in Southeast Asia [1,2]. In China, a large scale outbreak of HFMD emerged in 2007 in Shandong Province, with 1149 cases reported [3]. The nationwide epidemics of HFMD started in 2008 in Anhui province, with approximately 490,000 cases reported [4]. Since then, there has been a large outbreak of HFMD annually in China. In 2012, the Chinese Center for Disease Control and Prevention (China CDC) confirmed 2,168,737 cases in Mainland China including 569 deaths (published on the website of the Ministry of Health of China). HFMD has become an important public health issue in China.

Since HFMD was classified as a category C notifiable infectious disease by the Ministry of Health of China in 2008, the laboratory detection of EV71 and CVA16 has been routinely performed in pediatric sentinel sites set by the Infectious Disease Surveillance Center for HFMD patients. This allowed an extensive epidemiological and genetic characterization of the EV71 and CVA16 infection nationwide. However, the negative detection of EV71 and CVA16 had been reported frequently in China [5-7]. In few studies, other HEV serotypes were investigated, and suggested that CVA10, CVA6, CVA4 and CVA12 were associated with sporadic HFMD cases [6,7]. However, none of the aforementioned studies could ascertain their causal associations with HFMD development or disease severity of HFMD; due to lack of a four-fold antibody titer increase in convalescent samples, or make any comparison with healthy subjects. In addition, most previous data on HEV circulation have been reported from analysis of specimens from patients [7,8], whereas little epidemiologic information is available for the HEV circulation in healthy population, especially in China [9-11]. To address this query, a designed case–control study was performed to identify the HEV circulation in children with and without HFMD. Household distribution of HEVs was also investigated to disclose the epidemiologic characteristics of household distribution of HEVs in the healthy population.

## Methods

### Sample collection

The case–control study was performed in Jining city in Shandong province from May to October 2010. The children diagnosed as HFMD in Jining city were recruited into the study in the sentinel hospitals set by the national surveillance program for HFMD in Shandong province. The patients were identified according to the diagnostic criteria defined by the Ministry of Health (http://www.moh.gov.cn/publicfiles/business/htmlfiles/mohyzs/s3586/201004/46884.htm). Children with serious complications, including encephalitis, meningitis, acute flaccid paralysis, cardiorespiratory failure of death, were considered as severe HFMD. Children diagnosed as HFMD, but without any of the above mentioned serious complications, were classified as mild HFMD. Medical records of the HFMD patients were reviewed by physicians to collect the demographic data, the clinical symptoms and signs, laboratory findings, clinical diagnoses and outcomes.

Healthy children in Jining city were recruited as controls during the same period when the cases were recruited. Four villages that served as the sources of the patients in the selected hospitals were randomly selected as study sites. In each of two villages, one kindergarten was randomly selected to recruit healthy children less than 5-year-old. In other two villages, healthy children of less than 3-year-old were randomly recruited from families. Throat swabs were collected and personal information on demographic factors and medical history were obtained from their guardians by using a standard questionnaire. Throat swabs from parents who took care of healthy children and were willing to participate into the study in the two villages were collected simultaneously when sampling children. For the parents, personal information on demographic factors and medical history were collected via a questionnaire. All the recruited healthy children and parents did not exhibit HFMD-related symptoms before or at the time of sample collection.

This study was performed with the approval of the Ethical Committee of Beijing Institute of Microbiology and Epidemiology and Jining Infectious hospital and was conducted according to the principles expressed in the Declaration of Helsinki. At recruitment, written informed consent was obtained from all participants or guardians of pediatric participants.

### Detection and genotyping of HEV

For the detection of HEV, RNA were extracted from each specimen by using QIAamp® MinElute Virus Spin Kits (QIAGEN, Hilden, Germany) and the cDNA sample was synthesized by using SuperScript® III First-Strand Synthesis System for Reverse Transcription Polymerase Chain Reaction (RT-PCR) (Invitrogen, America). The detection of HEV and further classification of EV71 and CVA16 for HEV-positive samples were performed by real-time PCR using previously described primers, respectively [12]. To further identify the HEV serotypes other than EV71 and CVA16, semi-nested RT-PCR specific for a 5′ partial region of VP1 was performed for other HEV-positive samples by using previously reported primers [13]. The amplicons were subjected to sequencing and BLAST analysis.

### Sequence analysis of EV71

The VP1 sequences (891 bp) for EV71 positive samples from healthy children and HFMD patients were amplified by nested-PCR using primers (Table 1). The genomic sequences were assembled using Lasergene’s DNA SeqMan software (version 7.1.0, DNA Star Inc. Madison, WI, USA). The sequences obtained from the study were submitted to NCBI with the GenBank Accession Numbers: KF704042-KF704050, HQ668342, HQ668360, HQ668388, HQ668400, and HQ668414-HQ668422. The MEGA program (version 5.05, Sudhir Kumar, Arizona State University) was used for alignments and phylogenetic tree construction by neighbor-joining method or maximum likelihood method with 1000 bootstrap pseudo replicates. Similarities between strains were calculated by using BioEdit (version 7.13, http://www.mbio.ncsu.eud/bioedit/bioedit.html).

**Table 1:** Primers used for enterovirus 71 VP1 sequencing by RT-PCR

**Primer**	**Sequence**	**Position**	**Usage**
VP1-2382 F1	5′-ATAATAGCACTAGCGGCAGCCCA-3′	2382	Nested RT-PCR, 1st round
VP1-2415 F2	5′-ACCATGAAGTTGTGCAAGGA-3′	2415	Nested RT-PCR, 2st round
VP1-3387R2	5′-GCCCCAGACTGTTGTCCAAA-3′	3387	Nested RT-PCR, 2st round
VP1-3478R1	5′-GTCGCGAGAGCTGTCTTCCCA-3′	3478	Nested RT-PCR, 1st round
2833F	5′-GAGYTRTTCACCTACATGCG-3′	2833	Sequencing
3065R	5′-CTCGCRGGTGACATGAAYGG-3′	3065	Sequencing

### Statistical analysis

Descriptive statistics were performed, with continuous variables summarized as median and range, and categorical variables summarized as frequencies and proportions. The statistical significance of difference in HEV prevalence rates between various groups was tested using the *t* test for continuous variables and the *χ*^2^ test and Fisher’s exact test for categorical data. Analyses were performed using SPSS, version 11.5 (SPSS).

## Results

### Prevalence and serotypes of HEV in cases and controls

Altogether 579 HFMD patients were recruited into the study, with age ranging from 4 to 97 months (median, 27.5 months) and 395 (68.2%) were males. Among all the tested HFMD patients, 440 (76.0%) were infected with HEV, among whom the frequency of HEVs in severe HFMD patients was significantly higher than that in mild disease group, 82.1% (124/151) vs. 73.8% (316/428) (*P* = 0.04). Among 440 HEV positive samples from patients, 429 were successfully typed and 12 serotypes were identified: EV71 (253, 43.7%), CVA16 (104, 18.0%), CVA10 (33, 5.7%), CVA6 (22, 3.8%), CVA12 (6, 1.0%), echovirus 9 (3, 0.5%), echovirus 6 (2, 0.4%), CVB6 (2, 0.4%), CVA4 (1, 0.2%), CVA14 (1, 0.2%), echovirus 24 (1, 0.2%) and echovirus 3 (1, 0.2%). Respectively eight and eleven serotypes were determined from severe and mild HFMD patients. The EV71 (55.0% vs. 39.7%, *P* = 0.001) and CVA16 (11.9% vs. 20.0%, *P* = 0.024) positive rate differed significantly between severe and mild HFMD patients. For the rarely detected serotypes, CVA14 was only detected from severe cases, while echovirus 3, echovirus 24, CVB6 and CVA4 were only detected from mild cases. The frequencies of other five serotypes were evenly distributed between the two groups (all *P* > 0.05). The detailed distribution of HEV serotypes in HFMD patients is shown in Table 2.

**Table 2:** Prevalences of enterovirus serotypes in children with and without hand, foot, and mouth disease

	**HFMD cases**		**Healthy children (n = 254)**	***P *** **value**^**b**^	
	**Severe HFMD**	**Mild HFMD**		**Severe HFMD vs. mild HFMD**	**HFMD cases vs. healthy children**
	**(n = 151)**	**(n = 428)**			
HEV status					
Negative	27 (17.9)	112 (26.2)	195 (76.8)	0.04	< 0.001
Positive	124 (82.1)	316 (73.8)	59 (23.2)		
HEV serotypes^a^					
EV71	83 (55.0)	170 (39.7)	38 (15.0)	0.001	< 0.001
CVA16	18 (11.9)	86 (20.0)	7 (2.8)	0.024	< 0.001
CVA10	11 (7.3)	22 (5.1)	2 (0.8)	0.33	0.001
CVA6	4 (2.7)	18 (4.2)	4 (1.6)	0.39	0.09
E3	0	1 (0.2)	2 (0.8)	ND	0.17
E9	2 (1.3)	1 (0.2)	1 (0.4)	0.11	0.81
E24	0	1 (0.2)	1 (0.4)	ND	0.55
CVB6	0	2 (0.5)	1 (0.4)	ND	0.91
E6	1 (0.7)	1 (0.2)	0	0.44	ND
CVA12	2 (1.3)	4 (0.9)	0	0.68	ND
CVA4	0	1 (0.2)	0	ND	ND
CVA14	1 (0.7)	0	0	ND	ND
Untyped	2 (1.3)	9 (2.1)	3 (1.2)	ND	ND

^a^HFMD, hand, foot, and mouth disease; EV, enterovirus; CV, coxsackievirus; E, echovirus; NA; ND, not determined.

^b^Fisher exact test or *χ*^*2*^ test.

In total, 254 healthy children were included as control and had throat swabs collected. Their age ranged from 3 to 72 months (median: 48.0 month) and 136 (53.5%) were male. Altogether 59 (23.2%) of 254 throat swabs were found to be positive for HEV, and 56 HEV positive samples were successfully typed. Eight serotypes were determined with the most frequently presented serotypes as EV71 (38, 15.0%) and CVA16 (7, 2.8%), followed by CVA6 (4, 1.6%), CVA10 (2, 0.8%), echovirus 3 (2, 0.8%), echovirus 24 (1, 0.4%), echovirus 9 (1, 0.4%), and CVB6 (1, 0.4%).

In comparison with HFMD patients, the frequencies of HEVs in healthy controls were significantly lower, 76.0% (440/579) vs. 23.2% (59/254) (*P* < 0.001). The EV71 (43.7% vs. 15.0%, *P* < 0.001), CVA16 (18.0% vs. 2.8%, *P* < 0.001), and CVA10 (5.7% vs. 0.8%, *P* = 0.001) serotypes were significantly overrepresented in HFMD cases in comparison to healthy controls. The frequencies of other five serotypes (CVA6, echovirus 3, echovirus 9, echovirus 24, CVB6) were not significantly different between the two groups (all *P* > 0.05). Four HEV serotypes (echovirus 6, CVA12, CVA4 and CVA14) were only detected from HFMD cases, while not from healthy controls.

### Prevalence and serotypes of HEV among households

A total of 49 households involving 98 parents and 53 healthy children were recruited and sampled. The HEV infection rate for all family members was 19.2% (29/151). Children had significantly higher HEV infection rate than adults (28.3% vs. 14.3%, *P* = 0.037). The HEV infection rates were similar between mothers (12.24%; 6/49) and fathers (16.32%; 8/49) (*P* = 0.56). Twenty-two households (44.9%) had at least 1 family member with evidence of HEV infections, comprising seven serotypes: EV71 (11.92%), CVA16 (2.65%), CVA10 (1.32%), CVA6 (1.32%), echovirus 9 (0.66%), echovirus 3 (0.66%), and CVA4 (0.66%). In four households, both children and parents were infected with HEVs, although the virus serotypes involved were different within each family. The remaining 18 households had only children or parents with evidence of HEV infections. Among them, 10 households had CVA16, CVA6, E3, EV71, CVA10 infections in children, while 8 households had EV71, CVA16, CVA10, and CVA4 infections in parents (Table 3). There were 2 households (household 44 and 45) where both father and mother had HEV infections, and the same HEV serotype (EV71) was detected between father and mother. Among 4 households which had two children, siblings were all infected with EV71 in family 37, and siblings were all negative for HEV in other 3 households (households 27, 38 and 39).

**Table 3:** Circulation of enterovirus in healthy family members in Jining city, Shandong Province

**No.(%) of families (n = 49)**	**HEV status**		**HEV serotypes**^**a**^				
	**Children**	**Parents**	**Households**	**Child 1**	**Child 2**	**Father**	**Mother**
27 (55.1)	Negative	Negative	1-26	Negative	**—**	Negative	Negative
			27 (2 children)	Negative	Negative	Negative	Negative
10 (20.4)	Positive	Negative	28-36	EV71 (n = 3), CVA16 (n = 2), CVA6 (n = 2), CVA10 (n = 1), E3 (n = 1),	**—**	Negative	Negative
			37 (2 children)	EV71	EV71	Negative	Negative
8 (16.3)	Negative	Positive	38 (2 children)	Negative	Negative	EV71	Negative
			39 (2 children)	Negative	Negative	EV71	Negative
			40	Negative	**—**	CVA4	Negative
			41	Negative	**—**	Negative	EV71
			42	Negative	**—**	Negative	EV71
			43	Negative	**—**	Negative	EV71
			44	Negative	**—**	EV71	EV71
			45	Negative	**—**	EV71	EV71
4 (8.2)	Positive	Positive	46	EV71	—	CVA10	Negative
			47	CVA9	**—**	EV71	Negative
			48	EV71	**—**	CVA16	Negative
			49	EV71	**—**	Negative	CVA16

^a^EV, enterovirus; CV, coxsackievirus; E, echovirus.

### Genetic characterization of EV71 circulating in healthy children and HFMD patients

**Figure 1:**
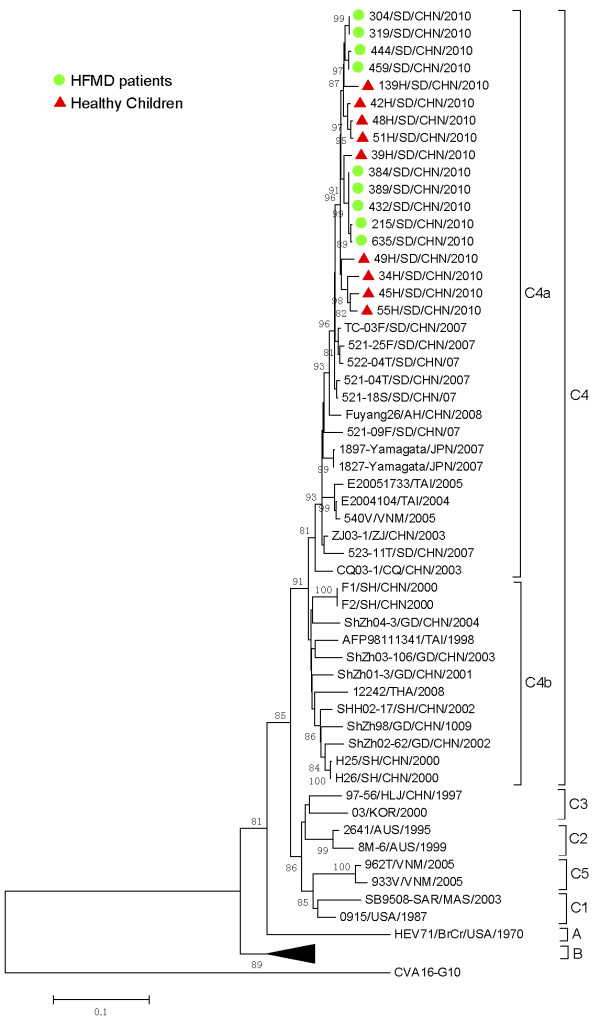
**Phylogenetic trees were constructed from the VP1 nucleotide sequences of EV71 using neighbor-joining method with 1000 bootstrap by CLC genomics workbench.** The tree was based on the 891 VP1 nucleotide sequences of EV71. The strains labeled with green dots were obtained from healthy children in our study. The strains labeled with red dots were obtained from HFMD patients in our study.

Among 38 EV71 positive specimens from healthy children, half (n = 19) were randomly selected and 9 entire VP1 sequences were successfully amplified and sequenced. Among 253 EV71 positive specimens from HFMD patients, 10% (n = 25) were randomly selected and 13 VP1 sequences were successfully amplified and sequenced. The failure to sequence EV71 VP1 in other specimens positive by RT-PCR may be ascribed to low viral load. The phylogenetic tree was constructed with the VP1 nucleotide sequences of both healthy children and HFMD patients from the present study and those downloaded from GenBank (Figure 1). All the sequenced EV71 strains in the current study were classified as belonging to the C4a group, demonstrating highest similarity with strains from Shandong province. The EV71 strains identified from healthy children and HFMD patients in this study showed sequence identity of 97.1%-100%. In addition, all isolates from members living in the same household (households 44 and 45 where both husband and wife had EV71 infection, and household 37 with all siblings infected with EV71) had 100% sequence homology, suggesting a possible interfamilial spread of this virus strain.

## Discussion

In this study, we identified a variety of HEV genotypes from throat swabs of HFMD patients in Jining, China. Our results show a diversified pathogen composition which is similar with reports from other areas of China [7,14-16], Spain [17], Korea [18], India [19] and Singapore [20]. By comparison between HFMD cases and healthy controls, we found that EV71, CVA16, and CVA10 serotypes were more frequently related to HFMD. In contrast, other five serotypes (CVA6, echovirus 3, echovirus 9, echovirus 24, CVB6) were detected in the two groups of subjects with comparable frequency. Some HEV serotypes were only detected from HFMD cases, and not from healthy controls. However, their roles cannot be confirmed due to the small number of positives found. In this study, CVA6 was detected infrequently whereas it has been associated with sporadic HFMD cases and outbreaks occurred recently in Japan [21], Spain [22], USA [23] and Thailand [24]. A study performed in Taiwan in 2010 documented CVA6 as a major cause of atypical HFMD, while CVA16 and EV71 were reported rarely [25]. In addition, we found that EV71 was overrepresented in severe HFMD patients, concurring with previous studies showing extremely high circulations of EV71 in more severe cases [26]. Our results also supported the notion that EV71 and CVA16 circulate widely and actively in China as two main causative agents of HFMD, as previously reported [6,27].

The aetiology of HFMD has changed with time occurred in China and in several other countries [15,16,24]. The sentinel surveillance studies performed in Linyi City, Shandong province, China, showed that the pathogen spectrum changed from 2008 to 2011, with the most prevalent HEV serotype being EV71 between 2008 to 2010, and CVA16 in 2011 [4,15,28]. CVA6 replaced CVA16 as the second most common serotype between 2010 to 2012 in Shenzhen, China [16]. In Thailand, HFMD is usually caused by EV71 and CVA16, but an outbreak of HFMD in 2012 was identified to be caused by CVA6 [24]. Considering that the population of the present study was restricted temporally and geographically, prolonged surveillance and more detailed molecular-typing surveillance of HEVs are needed to better understand the composite viral etiology of HFMD in this area.

Our understanding of epidemiological and genetic characteristics of HEV in the healthy population remains far from complete. In recent years, several studies among healthy individuals in different countries have shown diversity in HEV positive rates. Studies conducted among children in Shenzhen, China [29] and Norway [10,30,31] reported a HEV positive rate of 10.6 and 11.6% from stool samples respectively. Isolation rates of 64% and 35% were obtained from stool samples collected in children under 10-year-old and adults over 21-year-old in Mongolia [32]. With regard to EV71, diversity in positive rate has also been reported among healthy individuals in different countries. Han et al. reported positive rates of 0, 4.93 and 10.29% in throat swabs and 2.86, 3.94 and 8.82% in stools from three villages with different HFMD prevalence rate [9]. Studies conducted among children in Finland [33] and Norway [10,30,31] reported an EV71 positive rate of 0.3 and 1.4% from stool samples respectively, and isolation rate of 1.8% from stool samples were reported among children in Shenzhen, China [29]. Herein we demonstrated that 23.2% and 15.0% out of 254 healthy children carried HEV and EV71 respectively. This might represent the highest EV71 detection rate reported this far among healthy children. Our survey was performed during the local HFMD epidemic period ranging from May to August, which could partially explain the high EV71 frequency. The differences in specimens sampled, selection of subjects, and virus identification methods, as well as the climate, geography, crowding, and socio-economic status factors could also lead to the differences in the positive rate between our study and others.

EV71 is classified into three genotypes A, B and C, and within the genotypes B and C, there are further subgenotypes, B1-B5 and C1-C5. Recently, several studies also proposed that subgenotype C4 strains should be designated as a new genotype D and that the B5 isolates to be re-designated as B4 [34,35]. In China, the C4 subgenotype was identified as the most prominent circulating EV71 subgenotype [27]. All EV71 sequences reported in this study, regardless of disease status, cluster within the C4a clade, implying that C4a EV71strains predominated in Jining city in 2010. The large numbers of asymptomatic persons who carry HEVs, especially EV71, may serve as a reservoir for transmission of HEV to children and contribute to the large epidemics that occur annually.

Transmission of HEVs within a household is common. A recent prospective family cohort study investigated EV71 patients at a children’s hospital and family members of these patients who had EV71 has reported that the EV71 transmission rate to household contacts was 52%, and the transmission rate from children to parents was 41% [36]. Kuramitsu et al. found that interfamilial spread was responsible for 54% of non-polio HEV infections in healthy persons [32]. Our family study showed that 18.5% of family members were positive for HEV, and children had significantly higher HEV positive rate than adults. Limited transmission between parents and children was demonstrated, despite the inconsistent positive detection and incongruent serotypes obtained. Possible transmission between siblings and between husband and wife was suggested, however, only in few families. The information obtained in this study further supported the potential transmission of HEVs even among asymptomatic children, finally leading to a high reservoir for future epidemic. Our study findings stress the importance of personal hygiene to prevent infection with HEVs in the home environment. However, because of the limited number of families, further studies of larger sample size are needed. In addition, prospective follow-up of households would help clarify the distribution and transmission of HEVs within a household.

## Conclusions

In conclusion, this study demonstrated the co-circulation of multiple HEV serotypes in both HFMD cases and healthy children during HFMD epidemics. This study provides useful epidemiological data on the features of the spread of HEV among families as well. These findings have important public health implication for HFMD control, especially in HFMD high epidemic regions.

## Abbreviations

EV: Enteroviruses; HEVs: Human enteroviruses; HFMD: Hand, foot, and mouth disease; EV71: Enterovirus 71; CVA16: Coxsackievirus A16; RT-PCR: Reverse transcription polymerase chain reaction; E: Echovirus.

## Competing interests

The authors have no conflicts of interest to disclose.

## Authors’ information

Dr Zhang is an epidemiologist at the State Key Laboratory of Pathogen and Biosecurity, Beijing Institute of Microbiology and Epidemiology, Beijing, China. Her primary research interests include microbiology, epidemiology, and the genetic susceptibility to infectious diseases such enterovirus 71 infection and influenza.

## Pre-publication history

The pre-publication history for this paper can be accessed here:

http://www.biomedcentral.com/1471-2334/13/606/prepub

## References

[B1] Solomon T Lewthwaite P Perera D Cardosa MJ McMinn P Ooi MH Virology, epidemiology, pathogenesis, and control of enterovirus 71 Lancet Infect Dis 2010 13 11 778 790 10.1016/S1473-3099(10)70194-8 20961813

[B2] Yip CC Lau SK Woo PC Yuen KY Human enterovirus 71 epidemics: what’s next? Emerg Health Threats J 2013 13 19780 24119538 10.3402/ehtj.v6i0.19780 PMC3772321

[B3] Zhang Y Tan XJ Wang HY Yan DM Zhu SL Wang DY Ji F Wang XJ Gao YJ Chen L An outbreak of hand, foot, and mouth disease associated with subgenotype C4 of human enterovirus 71 in Shandong, China J Clin Virol 2009 13 4 262 267 10.1016/j.jcv.2009.02.002 19269888

[B4] Yang F Ren L Xiong Z Li J Xiao Y Zhao R He Y Bu G Zhou S Wang J Enterovirus 71 outbreak in the People’s Republic of China in 2008 J Clin Microbiol 2009 13 7 2351 2352 10.1128/JCM.00563-09 19439545 PMC2708525

[B5] He SJ Han JF Ding XX Wang YD Qin CF Characterization of enterovirus 71 and coxsackievirus A16 isolated in hand, foot, and mouth disease patients in Guangdong, 2010 Int J Infect Dis 2013 13 11 e1025 1030 10.1016/j.ijid.2013.04.003 23791223

[B6] Lu QB Zhang XA Wo Y Xu HM Li XJ Wang XJ Ding SJ Chen XD He C Liu LJ Circulation of Coxsackievirus A10 and A6 in hand-foot-mouth disease in China, 2009–2011 PLoS One 2012 13 12 e52073 10.1371/journal.pone.0052073 23272213 PMC3525556

[B7] Yang F Zhang T Hu Y Wang X Du J Li Y Sun S Sun X Li Z Jin Q Survey of enterovirus infections from hand, foot and mouth disease outbreak in China, 2009 Virol J 2011 13 508 10.1186/1743-422X-8-508 22054534 PMC3227625

[B8] Tseng FC Huang HC Chi CY Lin TL Liu CC Jian JW Hsu LC Wu HS Yang JY Chang YW Epidemiological survey of enterovirus infections occurring in Taiwan between 2000 and 2005: analysis of sentinel physician surveillance data J Med Virol 2007 13 12 1850 1860 10.1002/jmv.21006 17935170

[B9] Han J Ma XJ Xu WB Wan JF Li Q Tian C Gao C Wang M Tang LY Zhang Y EV71 viral secretion by symptomatic hand foot and mouth disease patients and their asymptomatic close contacts J Infect 2011 13 1 107 108 10.1016/j.jinf.2010.11.007 21087628

[B10] Witso E Palacios G Cinek O Stene LC Grinde B Janowitz D Lipkin WI Ronningen KS High prevalence of human enterovirus a infections in natural circulation of human enteroviruses J Clin Microbiol 2006 13 11 4095 4100 10.1128/JCM.00653-06 16943351 PMC1698346

[B11] Simonen-Tikka ML Hiekka AK Klemola P Poussa T Ludvigsson J Korpela R Vaarala O Roivainen M Early human enterovirus infections in healthy Swedish children participating in the PRODIA pilot study J Med Virol 2012 13 6 923 930 10.1002/jmv.23284 22499016

[B12] Verstrepen WA Bruynseels P Mertens AH Evaluation of a rapid real-time RT-PCR assay for detection of enterovirus RNA in cerebrospinal fluid specimens J Clin Virol 2002 13 Suppl 1 S39 S43 12091080 10.1016/s1386-6532(02)00032-x

[B13] Nix WA Oberste MS Pallansch MA Sensitive, seminested PCR amplification of VP1 sequences for direct identification of all enterovirus serotypes from original clinical specimens J Clin Microbiol 2006 13 8 2698 2704 10.1128/JCM.00542-06 16891480 PMC1594621

[B14] Xu M Su L Cao L Zhong H Dong N Xu J Enterovirus genotypes causing hand foot and mouth disease in Shanghai, China: a molecular epidemiological analysis BMC Infect Dis 2013 13 1 489 10.1186/1471-2334-13-489 24148902 PMC4015791

[B15] Zhang L Chen QY Liu H Tang LQ Mai HQ Emerging treatment options for nasopharyngeal carcinoma Drug Des Devel Ther 2013 13 37 52 23403548 10.2147/DDDT.S30753 PMC3565571

[B16] He YQ Chen L Xu WB Yang H Wang HZ Zong WP Xian HX Chen HL Yao XJ Hu ZL Emergence, circulation, and spatiotemporal phylogenetic analysis of coxsackievirus a6- and coxsackievirus a10-associated hand, foot, and mouth disease infections from 2008 to 2012 in Shenzhen, China J Clin Microbiol 2013 13 11 3560 3566 10.1128/JCM.01231-13 23966496 PMC3889734

[B17] Cabrerizo M Tarrago D Munoz-Almagro C Del Amo E Dominguez-Gil M Eiros JM Lopez-Miragaya I Perez C Reina J Otero A Molecular epidemiology of enterovirus 71, coxsackievirus A16 and A6 associated with hand, foot and mouth disease in Spain Int J Infect Dis 2013 in press 10.1111/1469-0691.12361 24033818

[B18] Park SH Choi SS Oh SA Kim CK Cho SJ Lee JH Ryu SH Pak SH Jung SK Lee JI Detection and characterization of enterovirus associated with herpangina and hand, foot, and mouth disease in Seoul, Korea Clin Lab 2011 13 11–12 959 967 22239028

[B19] Gopalkrishna V Patil PR Patil GP Chitambar SD Circulation of multiple enterovirus serotypes causing hand, foot and mouth disease in India J Med Microbiol 2012 13 Pt 3 420 425 22052995 10.1099/jmm.0.036400-0

[B20] Wu Y Yeo A Phoon MC Tan EL Poh CL Quak SH Chow VT The largest outbreak of hand; foot and mouth disease in Singapore in 2008: the role of enterovirus 71 and coxsackievirus A strains Int J Infect Dis 2010 13 12 e1076 e1081 10.1016/j.ijid.2010.07.006 20952237

[B21] Fujimoto T Iizuka S Enomoto M Abe K Yamashita K Hanaoka N Okabe N Yoshida H Yasui Y Kobayashi M Hand, foot, and mouth disease caused by coxsackievirus A6, Japan, 2011 Emerg Infect Dis 2012 13 2 337 339 10.3201/eid1802.111147 22304983 PMC3310456

[B22] Montes M Artieda J Pineiro LD Gastesi M Diez-Nieves I Cilla G Hand, foot, and mouth disease outbreak and coxsackievirus A6, northern Spain, 2011 Emerg Infect Dis 2013 13 4 676 678 10.3201/eid1904.121589 PMC3647425 23751014

[B23] Flett K Youngster I Huang J McAdam A Sandora TJ Rennick M Smole S Rogers SL Nix WA Oberste MS Hand, foot, and mouth disease caused by coxsackievirus a6 Emerg Infect Dis 2012 13 10 1702 1704 23017893 10.3201/eid1810.120813 PMC3471644

[B24] Puenpa J Chieochansin T Linsuwanon P Korkong S Thongkomplew S Vichaiwattana P Theamboonlers A Poovorawan Y Hand, foot, and mouth disease caused by coxsackievirus A6, Thailand, 2012 Emerg Infect Dis 2013 13 4 641 643 10.3201/eid1904.121666 23631943 PMC3647428

[B25] Huang WC Huang LM Lu CY Cheng AL Chang LY Atypical hand-foot-mouth disease in children: a hospital-based prospective cohort study Virol J 2013 13 209 10.1186/1743-422X-10-209 23800163 PMC3717056

[B26] Chang LY Lin TY Huang YC Tsao KC Shih SR Kuo ML Ning HC Chung PW Kang CM Comparison of enterovirus 71 and coxsackie-virus A16 clinical illnesses during the Taiwan enterovirus epidemic, 1998 Pediatr Infect Dis J 1999 13 12 1092 1096 10.1097/00006454-199912000-00013 10608631

[B27] Tan X Huang X Zhu S Chen H Yu Q Wang H Huo X Zhou J Wu Y Yan D The persistent circulation of enterovirus 71 in People’s Republic of China: causing emerging nationwide epidemics since 2008 PLoS One 2011 13 9 e25662 10.1371/journal.pone.0025662 21980521 PMC3181342

[B28] Yang F Du J Hu Y Wang X Xue Y Dong J Sun L Li Z Li Y Sun S Enterovirus coinfection during an outbreak of hand, foot, and mouth disease in Shandong, China Clin Infect Dis 2011 13 4 400 401 10.1093/cid/cir346 21785005

[B29] Wu W Xu WB Chen L Chen HL Liu Q Wang DL Chen YJ Yao W Li G Feng B Molecular identification and analysis of human enteroviruses isolated from healthy children in shenzhen, china from 2010 to 2011 PLoS One 2013 13 6 e64889 10.1371/journal.pone.0064889 23762262 PMC3675095

[B30] Witso E Palacios G Ronningen KS Cinek O Janowitz D Rewers M Grinde B Lipkin WI Asymptomatic circulation of HEV71 in Norway Virus Res 2007 13 1 19 29 10.1016/j.virusres.2006.07.015 16965832

[B31] Cinek O Witso E Jeansson S Rasmussen T Drevinek P Wetlesen T Vavrinec J Grinde B Ronningen KS Longitudinal observation of enterovirus and adenovirus in stool samples from Norwegian infants with the highest genetic risk of type 1 diabetes J Clin Virol 2006 13 1 33 40 10.1016/j.jcv.2005.03.007 15916916

[B32] Kuramitsu M Kuroiwa C Yoshida H Miyoshi M Okumura J Shimizu H Narantuya L Bat-Ochir D Non-polio enterovirus isolation among families in Ulaanbaatar and Tov province, Mongolia: prevalence, intrafamilial spread, and risk factors for infection Epidemiol Infect 2005 13 6 1131 1142 10.1017/S0950268805004139 16274512 PMC2870349

[B33] Honkanen H Oikarinen S Pakkanen O Ruokoranta T Pulkki MM Laitinen OH Tauriainen S Korpela S Lappalainen M Vuorinen T Human enterovirus 71 strains in the background population and in hospital patients in Finland J Clin Virol 2013 13 4 348 353 10.1016/j.jcv.2012.11.018 23261080

[B34] Li W Yi L Su J Lu J Zeng H Guan D Ma C Zhang W Xiao H Li H Seroepidemiology of human enterovirus71 and coxsackievirusA16 among children in Guangdong province, China BMC Infect Dis 2013 13 322 10.1186/1471-2334-13-322 23855481 PMC3717138

[B35] Yip CC Lau SK Lo JY Chan KH Woo PC Yuen KY Genetic characterization of EV71 isolates from 2004 to 2010 reveals predominance and persistent circulation of the newly proposed genotype D and recent emergence of a distinct lineage of subgenotype C2 in Hong Kong Virol J 2013 13 222 10.1186/1743-422X-10-222 23822185 PMC3716818

[B36] Chang LY Tsao KC Hsia SH Shih SR Huang CG Chan WK Hsu KH Fang TY Huang YC Lin TY Transmission and clinical features of enterovirus 71 infections in household contacts in Taiwan JAMA 2004 13 2 222 227 10.1001/jama.291.2.222 14722149

